# TRANSCRIPTOMIC SIGNATURES OF AGING, GENETICS, AND MORTALITY RISK: FINDINGS FROM THE LONG LIFE FAMILY STUDY

**DOI:** 10.1093/geroni/igad104.1968

**Published:** 2023-12-21

**Authors:** Mary Wojczynski, Nancy Glynn, Nalini Raghavachari

## Abstract

The Long Life Family Study (LLFS), funded by the National Institute on Aging, is an international collaborative study of the genetics and familial components of exceptional longevity and healthy aging. We phenotyped 4,953 individuals from 539 two-generational families (1,727 proband; 3,226 offspring) at baseline (2006-2009). A second visit (2014-2017) was conducted for 2,904 (478 proband; 2,426 offspring) participants, with a third visit ongoing. The longitudinal, comprehensive in-person visits measured domains of healthy aging, including physical performance, cognition, and blood markers. Extensive genetic analyses were performed using the baseline blood draw, including genotyping with the Illumina 2.5M Human Omni array, linkage analyses with the families, whole genome sequencing using the TopMED protocol, metabolomic assays, and trascriptomics (RNA-seq). Collectively, this symposium will present novel findings that examined differences in gene expression between those of extreme old age compared to younger participants, elucidate potential new genomic regions and variants associated with ankle brachial index, examine rare variants under linkage peaks for perceived physical fatigability, and compare mortality and cause of death between LLFS and the US population. Specifically, Ms. Li will share results on gene expression profiles in aging. Then, Ms. Ressler will share findings on the genetics of ankle brachial index with linkage and association results. Next, Ms. Gay will discuss associations under the linkage peak for perceived physical fatigability. Lastly, Ms. Yao will present mortality and patterns of death in the LLFS. As Discussant, Dr. Nalini Raghavachari from the NIA will provide insights and propose future directions for LLFS.

